# Are we *Lovin’ It*? A single blind randomised controlled trial to investigate the influence of high in fats, salt and sugar food brand advertising on cognitive, affective and behavioural intention outcomes in UK adults

**DOI:** 10.1017/S1368980026103115

**Published:** 2026-07-20

**Authors:** Christus Ferneyhough, Rebecca K. Evans, Amy Finlay, Katharine Jenner, Emma Boyland

**Affiliations:** 1 Obesity Health Alliance, UK; 2 Department of Psychology, University of Liverpool, UK

**Keywords:** Randomised controlled trial, brand advertising, HFSS food

## Abstract

**Objectives:**

The effectiveness of food advertising restrictions may be limited by the allowance of brand advertising. This study investigated the impact of brand advertising on cognitive, affective and behavioural intention outcomes for food products high in fat, salt and/or sugar (HFSS).

**Design:**

Online randomised controlled trial. Participants were randomly allocated to one of three groups: HFSS brand advertisements, non-HFSS brand advertisements or non-food brand advertisements (control). Eight images of brand advertisements were individually presented via an online research platform. The primary outcomes were purchase intention and craving, measured using 100-point slider scales. Secondary outcomes were brand recognition, recall and product associations. Generalised linear models examined craving and purchase intention for HFSS foods. Group differences in brand recognition, recall and product associations were tested using Kruskal–Wallis and ANOVA.

**Setting:**

Online.

**Participants:**

UK adults (*N* 191) aged 18–87 (*M* = 47; sd = 16·15).

**Results:**

The mean composite purchase intention score for the HFSS group (234·89) was not significantly different (*p* > 0·05) from coefficients for non-HFSS (−51·91) and non-food groups (31·21). Similarly, no significant group differences were found for cravings. The HFSS group was significantly more likely to recognise, recall and associate more products with viewed brands, compared to non-HFSS and non-food groups (*p*s < 0·01).

**Conclusion:**

Exposure to HFSS brand advertising triggered recognition, recall and product associations with viewed brands. However, this did not translate into increased craving or purchase intention for HFSS foods. Future research should examine the effects of brand advertising in real-world settings.

## Introduction

The marketing of unhealthy food has been recognised as a key driver of poor dietary quality and associated negative health outcomes globally^(1)^. Regardless of the setting, unhealthy food products and brands are more frequently advertised than healthier products and brands^(2–5)^, which contributes to skewed social norms surrounding the extent that these products should be included in the typical diet^(6)^. While fast food and soft drinks are frequently advertised, UK Public Health guidance states that such foods and beverages (hereafter: foods) that are high in fat, salt and sugar (HFSS) should be eaten infrequently and in small amounts^(7)^.

Due to the public health challenges associated with food marketing, in 2010, the WHO recommended that restrictions to food marketing be enforced among member states^(8)^. In 2023, additional guidance stated these restrictions should be mandatory and protect children of all ages^(9)^. In the UK Government’s Obesity Strategy published in 2020^(10)^, a ban on advertising of HFSS foods before 9pm on television was proposed, alongside a ban on paid-for HFSS food advertising online. These restrictions were planned for 2022, but implementation was delayed three times, to January 2026^(11)^.

It is becoming increasingly important to consider advertising impacts through other advertising formats as when restrictions are implemented on television and online, marketing will likely be displaced to less restricted settings^(12)^. Outdoor spaces may be a key area of interest, as marketing companies suggest that outdoor advertising is more affordable for businesses compared to television, online or audio and still yields significant returns^(13,14)^. This is supported by a report from the Food Foundation showing that in the time since television and online restrictions were first proposed, outdoor advertising spend by food companies has increased by 28 %^(15)^.

Despite the lack of National-level outdoor marketing policy, positive progress has been made regarding outdoor regulations in the UK at a local level. By December 2024, twenty-one local authorities and Transport for London had implemented healthier food advertising policies^(16)^. Significant reductions in unhealthy household food purchases were observed following implementation of the Transport for London policy^(17)^; however, no significant changes in self-reported exposure to or consumption of unhealthy food products were observed following the implementation of a similar outdoor advertising policy in Bristol^(18)^. It is possible that effects of these restrictions would have been greater if (1) all outdoor food marketing spaces were in scope of the policy and (2) brands synonymous with unhealthy foods could not advertise their brand at all.

Brand advertising (i.e. a brand advertising their logo without an identifiable product) is frequently observed outdoors in the UK^(19)^, with recent evidence finding that almost 40 % of food advertisements were for brands rather than specific products^(15)^. This form of marketing is exempt from both existing and planned restrictions which instead assess individual products that can or cannot be marketed based on their nutritional quality^(20)^. Brands often develop a unique identity to separate themselves from competitors, which allows them to connect emotionally with consumers^(21)^. Evidence suggests that the development of a brand ‘personality’ can increase the popularity of food brands, specifically with young adults^(22)^, while branding alone has been shown to elicit neural responses both in children^(23)^ and adults^(24)^. Despite this, research into the effect of brand advertising is limited. A systematic review found that exposure to brand marketing may result in cognitive and behavioural outcomes (preference, intended consumption, choice and intake)^(25)^. However, this was not consistently found across the nineteen studies included, most of which used food packaging as a form of marketing exposure. With an observed increase in outdoor HFSS brand advertising following restrictions to HFSS product advertising^(15)^, it is important to understand the potential impacts of this advertising on cognitive, affective and behavioural intention outcomes.

Therefore, this study aimed to explore the effect of HFSS brand advertising exposure (compared to non-HFSS brand advertising and non-food brand advertising) on craving and purchase intention for HFSS food products. Secondary aims were to examine differences in brand recognition, recall and product associations across advertising conditions (HFSS, non-HFSS and non-food), based on participants’ responses to the brands they were exposed to.

We hypothesised that HFSS brand advertising would have the greatest impact on craving and purchase intention relative to non-HFSS or non-food brand advertising and would trigger greater brand recognition, recall and more product associations than exposure to non-HFSS or non-food brands.

## Methods

### Study design

The study utilised a three-arm, randomised controlled trial design, hosted on the online research platform, Qualtrics. Ethical approval (Ref: 12430) was granted by the University of Liverpool Institute of Population Health Research Ethics Committee in April 2023.

### Participant sample

**Figure 1.**
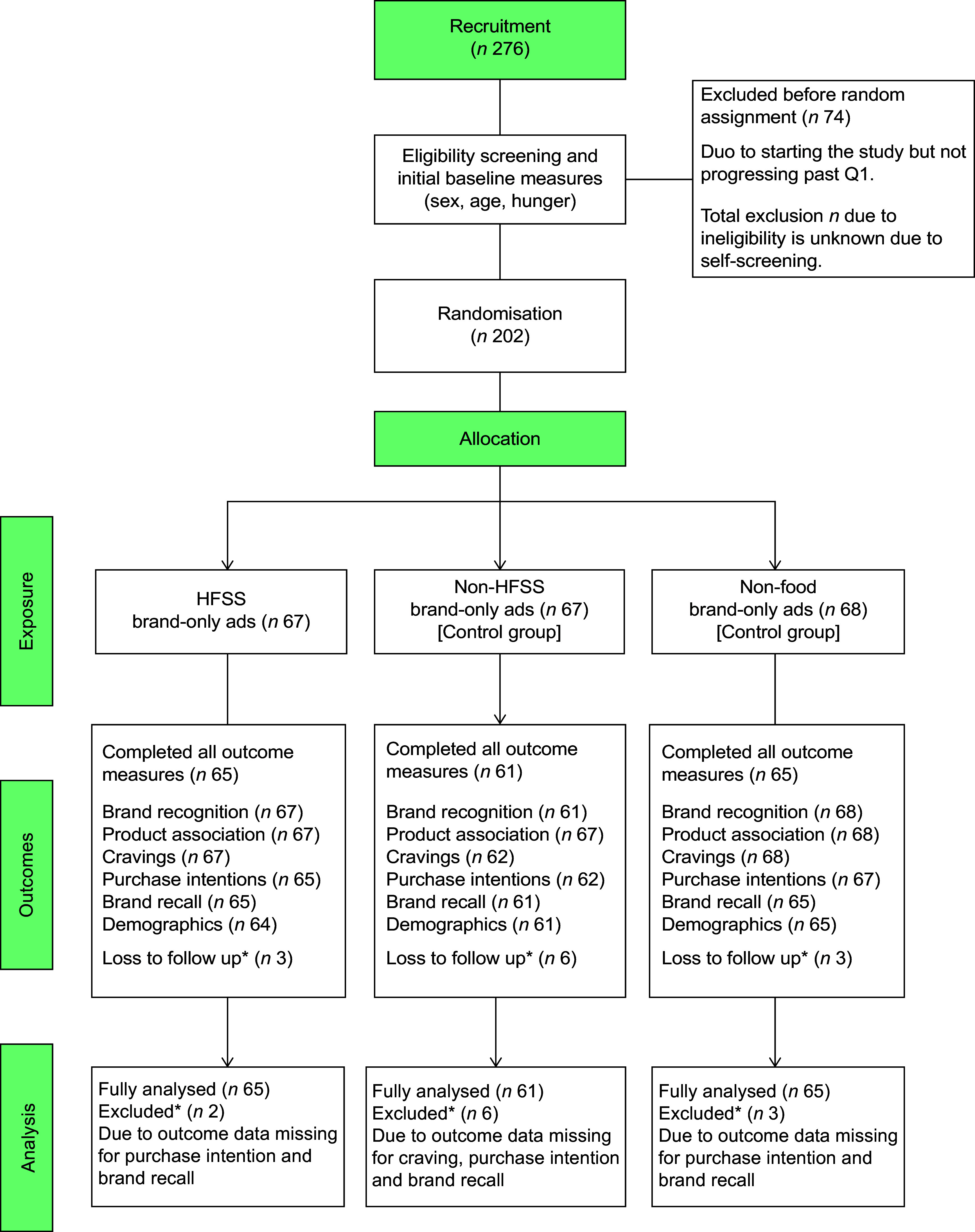
Consort flow diagram. *Loss to follow-up refers to participants that did not complete all questions. **Excluded refers to participants who were not included in the statistical analysis (only descriptives).

A total of *N* 276 participants were recruited to the study, and *N* 202 were randomised to a study condition. *N* 11 participants had missing data for primary and/or secondary outcomes; the data for these participants were retained for descriptive purposes, but only complete cases were included in statistical analyses (*N* 191). A consort flow diagram displaying the numbers of participants at each stage of the study is available in Figure 1. Participants were adults living in the UK who identified themselves as eligible according to the inclusion and exclusion criteria presented in Table 1. Individuals with medically restrictive diets were ineligible to take part due to predetermined dietary choices which may have introduced significant heterogeneity into responses.

**Table 1.** Participant inclusion and exclusion criteria Table 1 long description.

Inclusion criteria	UK adults (≥18) with access to the internet Able to provide informed consent
Exclusion criteria	Individuals with medically restrictive diets (pregnant and breastfeeding women, diabetics, Coeliac disease) Individuals who cannot read written English

Participants were recruited through social media posts containing a study link or QR code to a study ‘investigating how the environment impacts people’s product perceptions and responses’. In effort to minimise selection bias, social media posts were posted on local community groups based on geography rather than interests or topics. Prospective participants were asked to read through the eligibility criteria and certify that they met each of the criteria. Participants were randomly allocated (via Qualtrics) to one of three groups with a ratio of 1:1:1. The groups were as follows: (1) HFSS brand advertising, (2) non-HFSS brand advertising and (3) non-food brand advertising. Participants were blind to the group they were allocated and to the true aims of the study.

## Materials and procedure

### Brand selection

To maximise the likelihood that brands would be familiar to participants in all exposure groups, brands used in the study were (1) listed in the top 100 UK brands by annual revenue^(26)^ or (2) had 90 %+ recognition in a national You. Gov (Q3 2022) survey^(27)^. Food brands were deemed HFSS if the brand or brand name was synonymous or inextricably linked to a specific HFSS product, in line with WHO guidance^(28)^. Food brands were deemed non-HFSS if they were synonymous with products that did not meet HFSS criteria and were not linked to specific HFSS products.

### Stimuli

Participants saw eight images of HFSS, non-HFSS or non-food brand-only advertising on a range of outdoor advertisement structures (see all *n* 24 advertisements in online supplementary materials). All images were digitally altered by the research team so each advertisement featured only a non-text-based logo with no product images. This excluded brands where the logo consisted of the brand name or features recognisable packaging (e.g. a bottle).

**Figure 2.**
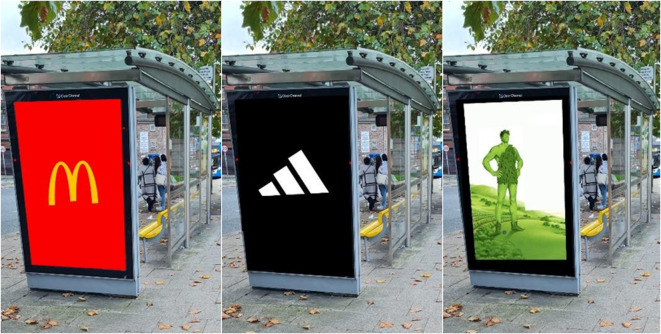
Example advertisement from each exposure group (left: high in fats, salt and sugar, middle: non-food, right: non-high in fats, salt and sugar).

The brand advertisements were digitally imposed onto researcher-photographed images of outdoor advertising spaces consisting of bus shelters, telephone boxes and billboards in Liverpool, England. An example from each exposure group is shown in Figure 2. The full list of included brands for each group along with the brand-only advertising images which formed the visual stimuli is included in Supplementary material A.

### Procedure

Prior to beginning the study, participants were asked to read the full study information sheet and electronically provide their consent to taking part in the study. At baseline, participants were asked for their sex, age and their level of perceived hunger. These variables were used to characterise the sample and were tested as covariates in primary analyses. To disguise the aims of the study, participants were asked three dummy questions relating to their mood (perceived happiness, stress and tiredness). Participants in each of the three study groups were then shown eight brand advertisements (as defined above). Advertisements were shown one at a time, with participants able to look through the eight pages with no set time limit to exposure. During exposure, participants completed measures of brand recognition and product association.

**Figure 3.**
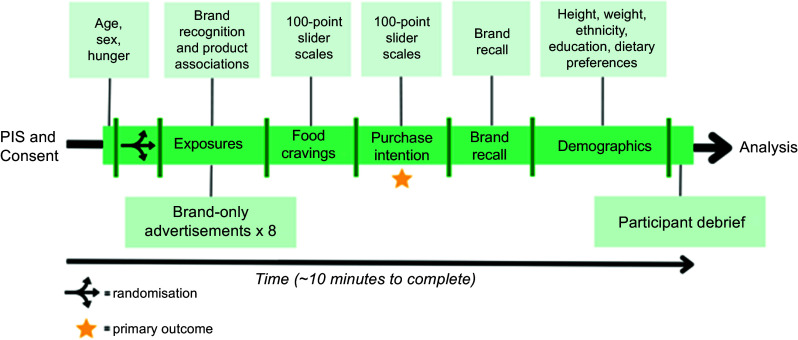
Survey flow.

Following exposure, participants completed measures of craving, purchase intention and brand recall. Finally, participants reported their ethnicity, highest level of education achieved, dietary preferences and height and weight (used to calculate BMI). These variables were used to characterise the sample and were tested as covariates in primary analyses. The survey flow is displayed in Figure 3.

### Outcomes

The primary outcome variables were craving and purchase intention. Secondary outcomes were brand recognition, brand recall and product associations.

#### Craving and purchase intention

Following advertising exposure, participants reported the extent to which they agreed with statements regarding their current cravings (‘I am craving this product right now’) for ten individual HFSS product types, using 100-point slider scales (anchored 0 = *strongly disagree to* 100 = *strongly agree)*. The ten product types listed mirror those in scope of pending HFSS advertising legislation [Restrictions on the Advertising of Less Healthy Food Regulations 2025]^(29)^ such as chocolate, pizza and crisps. Composite scores of craving (0 to 1000) were calculated for each participant. This process was repeated to measure purchase intention, with the statement ‘I intend to buy this food the next time I go shopping’. Question statements and scales were adapted and abbreviated from previous studies measuring food craving^(30)^ and purchase intention^(31)^. Higher scores indicate greater craving and purchase intention.

#### Brand recognition and product association

Brand recognition was measured using an adapted version of the Australian Brand Awareness Instrument^(32)^. Participants were asked to name the brand featured in the advertisement on their screen. In anticipation of incorrect spelling or abbreviation of brands, a scoring system was utilised, including a ‘partially correct’ category. The scoring system and list of participant responses are in Supplementary Material B, showing answers interpreted as correct (two points), partially correct (one point) and incorrect (zero points). Cumulative point totals for the eight brand exposures (zero to sixteen points) were recorded for each participant.

Whilst viewing the brand advertisement, participants were asked to list up to three products that they associate with the brand. General categorical descriptions (e.g. ‘burger’, ‘shoes’, ‘vegetables’) and specific product names (e.g. ‘Big Mac’, ‘Air Max’, ‘Sweetcorn’) were accepted as correct associations. If a participant provided overlapping general/specific answers (e.g. ‘burger’, ‘Big Mac’), both associations were considered correct. Non-product associations (e.g. ‘cheap’) were considered incorrect associations. Spelling errors were ignored if the intent was clear. Cumulative correct product associations were recorded for each participant (zero to twenty-four) and explored descriptively.

#### Brand recall

Participants were asked to recall the brands they were exposed to at the start of the survey. To report brands, participants were provided with eight open text boxes. The total number of brands recalled (zero to eight) was recorded.

### Sample size calculation

The required sample size was calculated using OpenEpi^(33)^. Effect size estimates were informed by previous findings of Karremans et al.,^(34)^ who demonstrated that subliminal priming significantly influences brand choice. The *percent of unexposed with outcome* was set at 30 %, and the *percent exposed with outcome was* set at 54 %. Calculations suggested that to detect similar-sized effects to Karremans et al., in the present study with 80 % power and a significance level of 0·05 would require a minimum of *N* 148 participants. We planned to recruit over this sample size by 20 % to account for participant attrition and methodological contrasts to the reference study. Resultantly, the required sample was *N* 178. For full power analysis, see Supplementary Material C.

### Data analysis

This study was not pre-registered, so analysis should be treated as exploratory.

Statistical analysis was completed in R^(35)^. Only participants who completed the study in full were included in statistical analyses. There were no discernible differences in demographic profiles or group allocation between the excluded and included samples, suggesting attrition was not systematic and did not bias one specific intervention arm. For all analyses, results were deemed significant at *P* < 0·05.

Descriptive analysis explored the distribution of continuous (age, BMI, hunger) and categorical (sex, ethnicity, level of education, dietary preferences) participant characteristic variables between groups.

#### Primary analysis

Craving and purchase intention variables were non-normally distributed and showed non-linear relationships with exposure; therefore, three regression approaches were tested: a generalised linear model, a square-root–transformed model and a beta regression. Across models, *p*-values and predicted means were comparable and none showed significant effects. We opted to present the bivariate generalised linear models in the Results section for interpretation, and the other models are presented as sensitivity analyses in Supplementary Material D. Specifically, the generalised linear regression models explored associations between advertising exposure and craving and purchase intention for HFSS foods. Several participant variables (hunger, age, sex, BMI, education, ethnicity, dietary preferences) were tested as covariates. Multivariable linear regressions were then used to generate models adjusted for covariates which were statistically significant in the bivariate models.

#### Secondary analysis

Histograms and Shapiro–Wilk tests revealed that brand recognition and brand recall were not normally distributed. Consequently, group differences in these outcomes were analysed with the non-parametric Kruskal–Wallis test. Where significant differences were observed, pairwise between comparisons using post hoc Dunn’s tests with Bonferroni correction were computed.

Group differences in product associations (normally distributed) were compared using an ANOVA followed by Tukey’s post hoc test. Additionally, simple descriptive statistics were presented for product associations for each brand individually.

## Results

### Participant characteristics

Of 276 recruited participants, 74 (26 %) were excluded prior to randomisation for not completing survey questions beyond providing consent. Of the 202 participants randomised (see Table 2), eleven (5 %) were excluded due to missing primary and/or secondary outcome data. This left a final analytic sample of 191 participants. No missing data were present in the final analytic sample.

**Table 2.** Characteristics of randomised participants by group Table 2 long description.

	High in fats, salt and sugar (*n* 67)		Non-high in fats, salt and sugar (*n* 67*)*		Non-food (*n* 68)	
Characteristics	*n*	%	*n*	%	*n*	%
**Sex**						
Male	16	24	19	28	17	25
Female	51	76	48	72	51	75
Age in yrs, mean (sd)	47·1	16·9	47·8	16·2	46·9	15·6
Baseline hunger, mean (sd)	20	3	21	6	17	3
**BMI**						
BMI in kg/m^2^, mean (sd)	30·1	11·2	29·9	12·6	27·5	8·6
Underweight (<18·5 kg/m^2^)	1	1·6	3	5·1	1	1·6
Healthy weight (≥18·5 & <25 kg/m^2^)	23	37·8	24	40·7	29	46·0
Overweight (≥25 &<30 kg/m^2^)	20	32·8	18	30·5	18	28·6
Obesity (≥30 kg/m^2^)	17	27·8	14	23·7	15	23·8
BMI missing values	3		6		3	
BMI anomalies removed*	3		2		2	
**Ethnicity**						
White	57	89	58	95	56	86
Asian or Asian British	4	6	0	0	4	6
Black, Black British, Caribbean, or African	2	3	1	2	0	0
Mixed or multiple ethnic groups	1	2	2	3	3	5
Other	0	0	0	0	2	3
Ethnicity missing values	3		6		3	
**Education**						
Primary education	1	1·6	0	0	0	0
Secondary education	21	32·8	17	27·9	16	24·6
Undergraduate degree	26	40·6	31	50·8	23	35·4
Masters degree	15	23·4	7	11·5	18	27·7
Doctorate	1	1·6	6	9·8	8	12·3
Education missing values	3		6		3	
**Dietary preferences**						
None	56	87·5	52	85·2	54	83·1
Vegetarian	3	4·7	3	4·9	3	4·6
Pescatarian	1	1·6	3	4·9	3	4·6
Vegan	2	3·1	0	0	1	1·5
Religious dietary practices	2	3·1	1	1·6	2	3·1
Other	0	0	2	3·3	2	3·1
Dietary preferences missing values	3		6		3	

* Implausible BMI values removed were <12 and >70 kg/m^2^.

#### Purchase intention

Bivariate regressions indicated that there was no significant difference in purchase intention for HFSS foods between the HFSS (median = 218, interquartile range [IQR] = 65–350), non-HFSS (median = 128, IQR = 53–300) and non-food (median = 190, IQR = 104–399) brand advertising exposure groups (*p*s >.05). Increasing age was significantly associated with reduced purchase intention (−4·47; 95 % CI, −6·03, −2·91, *p* < 0·01) whereby, on average, each additional year of age reduced the purchase intention score by 4·47. White ethnicity was also significantly associated with reduced purchase intention (−100·76; 95 % CI, −187·34, −13·18; *p* < 0·01). Associations with other variables were NS. See Table 3.

**Table 3.** Bivariate associations of exposure and participant characteristics with purchase intention for high in fats, salt and sugar foods Table 3 long description.

Variable	Level	Coefficient	se	95 % CI	*P*-value
**Advertising exposure**	high in fats, salt and sugar (Intercept)	234·89	23·24	189·34, 280·44	
	Non-high in fats, salt and sugar	−51·91	33·53	−117·63, 13·81	0·123
	Non-food	31·21	32·87	−33·20. 95·63	0·343
**Age**		−4·47	0·80	−6·03, −2·91	<0·01*
**Baseline hunger**		0·59	0·54	−0·47, 1·65	0·276
**Sex**	Female (Intercept)	218·01	15·92	186·80, 249·23	
	Male	43·03	31·29	−18·29, 104·35	0·171
**BMI**	Healthy weight (≥18·5 & <25 kg/m^2^) (Intercept)	215·43	21·30	173·68, 257·19	
	Underweight (<18·5 kg/m^2^)	−14·86	73·35	−158·63, 128·90	0·840
	Overweight (≥25 & <30 kg/m^2^)	11·23 (32,71)		−52·87, 75·33	0·732
	Obesity (≥30 kg/m^2^)	30·25	33·62	−35·63, 96·14	0·369
**Education**	Masters or doctorate (Intercept)	226·8	25·01	177·77, 275·83	
	Primary or secondary education	−12·20	25·01	−81·53, 57·13	0·731
	Undergraduate degree	7·238	32·49	−56·45, 70·92	0·824
**Ethnicity**	Non-white (Intercept)	317·00	41·91	234·86, 399·14	
	White	−100·76	44·18	−187·34, −13·18	0·024*
**Dietary preference**	None ([Intercept)	232·25	14·51	203·82, 260·68	
	Specific dietary preference	−40·25	37·79	−114·31, 33·92	0·288

Education, ethnicity and dietary preference groups condensed as original categories had < 5 data points.

* Statistically significant (*p* < 0·05).

A multivariable model (adjusted for age and White ethnicity) demonstrated that the association between advertising exposure and purchase intention remained non-significant. See Supplementary Material Appendix E, Table 3 for detail.

#### Craving

Bivariate regressions indicated that there was no significant difference in craving for HFSS foods between the HFSS (median = 196, IQR = 60–343), non-HFSS (median = 155, IQR = 64–331) and non-food (median = 141, IQR 21–383) advertising exposure groups (*p*s >.05). Being male was a significant predictor of increased craving (108·58; 95 % CI, 44·79, 172·35; *p* < 0·01). Similarly, baseline hunger was associated with increased craving (1·54; 95 % CI, 0·43, 2·65; *p* < 0·01), where each one-unit increase in hunger score resulted in an increased craving score of 1·54, on average. Conversely, increasing age was associated with reduced craving (−4·43; 95 % CI, −6·00, −2·63; *p* < 0·01). See Table 4.

**Table 4.** Bivariate associations of exposure and participant characteristics with craving for high in fats, salt and sugar foods Table 4 long description.

Variable	Level	Coefficient	se	95 % CI	*P*-value
**Advertising exposure**	high in fats, salt and sugar (Intercept)	233·65	25·14	184·38, 282·91	
	Non-high in fats, salt and sugar	−11·49	36·7	−82·57, 59·60	0·752
	Non-food	−12·73 (35·55)		−82·40, 56·94	0·721
**Age**		−4·32	0·86	−6·00, −2·63	<0·01*
**Baseline hunger**		1·54	0·56	0·43, 2·65	<0·01*
**Sex**	Female (Intercept)	197·53	16·56	165·0, 230·00	
	Male	108·58	32·54	44·79, 172·35	<0·01*
**BMI**	Healthy weight (≥18·5 & <25 kg/m^2^) (Intercept)	213·58	23·5	167·44, 259·71	
	Underweight (<18·5 kg/m^2^)	21·01	81·06	−137·87, 179·88	0·796
	Overweight (≥25 & <30 kg/m^2^)	38·12	36·14	−32·67. 109·00	0·292
	Obese (≥30 kg/m^2^)	−4·15	37·15	−76·96, 68·65	0·911
**Education**	Masters or doctorate (Intercept)	269·34	27·43	215·57, 323,10	
	Primary or secondary education	−60·25	38·80	−136·29, 15·8	0·122
	Undergraduate degree	−65·10	35·64	−134·95, 4·74	0·069
**Ethnicity**	Non-white (Intercept)	288·17	46·76	196·52, 379·82	
	White	−70·76	49·29	−167·37, 25·85	0·153
**Dietary preference**	None (Intercept)	224·07	16·10	192·51, 255·63	
	Specific dietary preference	2·78	41·94	−79·41, 85·00	0·947

Education, ethnicity and dietary preference groups condensed as original categories had <5 data points.

* Statistically significant (*p* < 0·05).

The multivariable model (adjusted for age, baseline hunger and sex) showed that the association between advertising exposure and craving remained non-significant. See Supplementary Material Appendix E, Table 4 for detail.

#### Brand recognition

**Figure 4.**
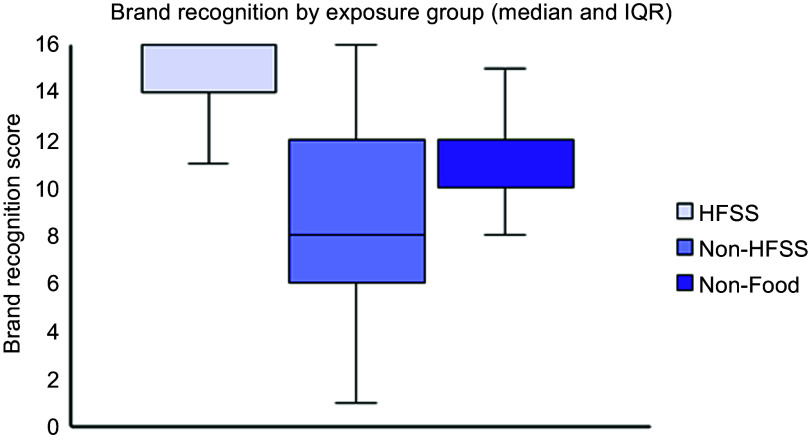
Boxplots to show median brand recognition scores for each group. *Median lines are not visible for high in fats, salt and sugar and non-food groups due to overlap with the upper quartile.

There was a statistically significant (H(2), 75·8; *p* < 0·01) difference in brand recognition scores between advertising exposure groups. The HFSS group correctly recognised significantly more brands (median = 14, IQR = 14–16) compared with the non-HFSS (median = 8, IQR = 6–12, Z, 8·28; *p* < 0·01) and non-food (median = 12, IQR = 10–12, Z, 5·36, *p* < 0·01) groups. The non-food group also recognised significantly more brands than the non-HFSS group (Z, 2·95, *p* < 0·01). See Figure 4.

#### Product associations

**Figure 5.**
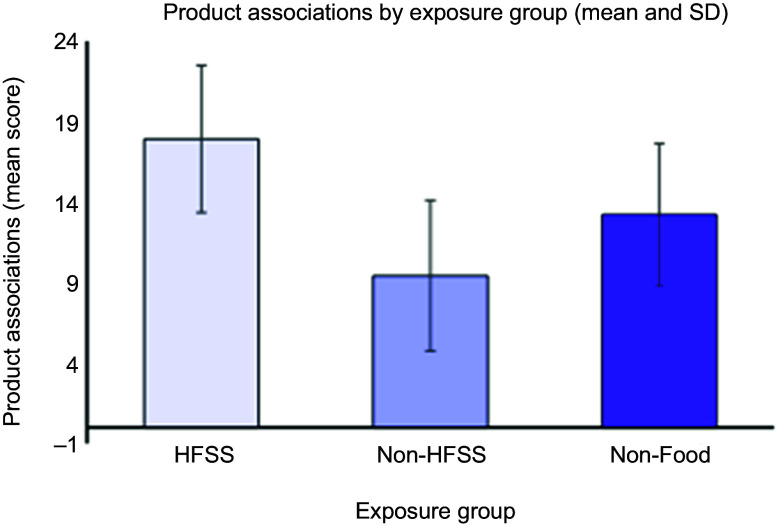
A bar chart to show mean product associations for each group.

**Figure 6.**
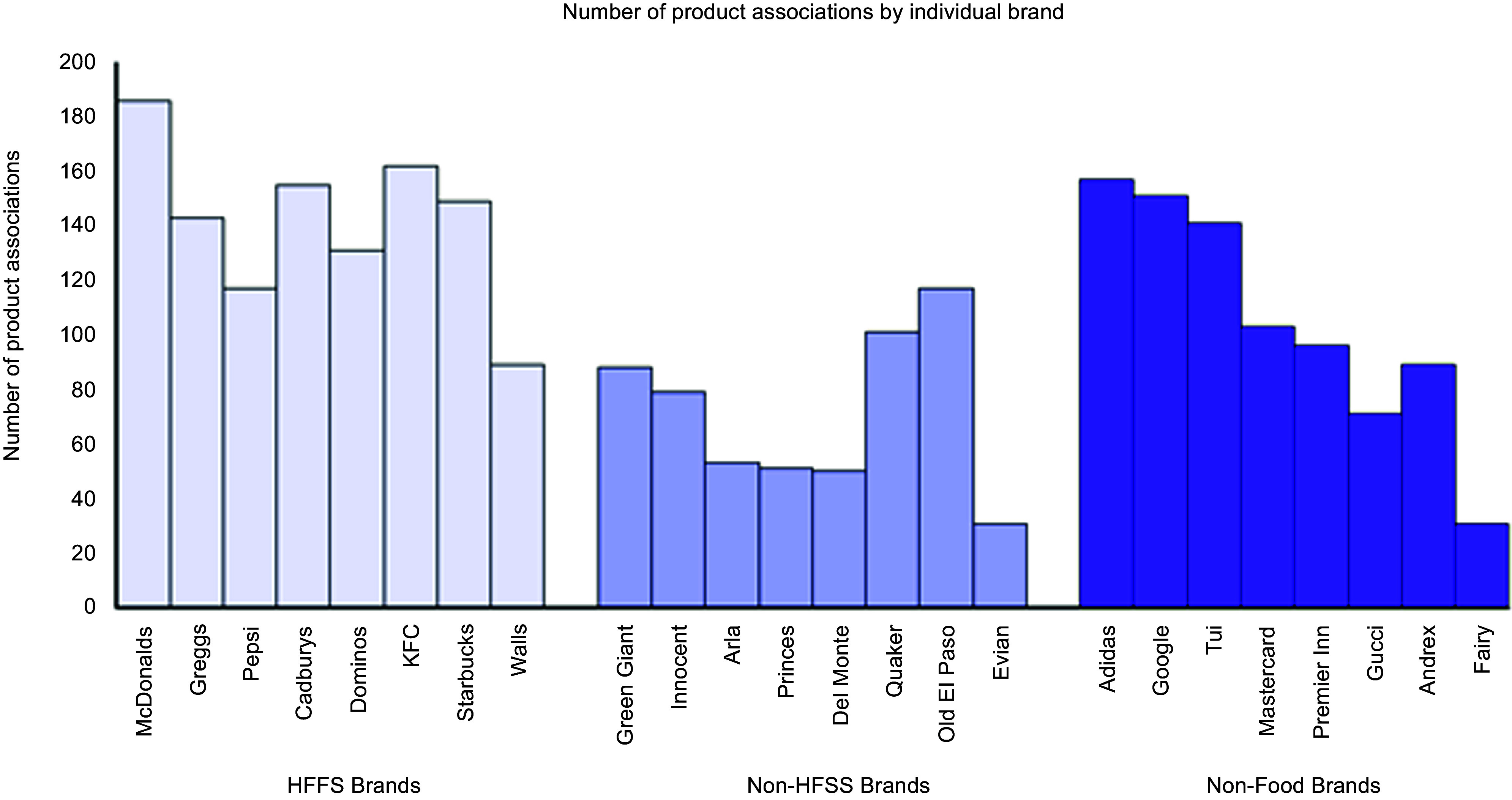
A bar chart showing the total number of product associations for each individual brand.

Product association scores between groups were significantly different (*F*(2, 199) = [57·78], *p* < 0·01). Compared to the HFSS group, participants recalled fewer products associated with the brands in the non-HFSS (−8·45; 95 % CI, −10·30, −6·59; *p* < 0·01) and non-food groups (−4·69; 95 % CI, −6·54, −2·84; *p* < 0·01). Participants in the non-food group recalled significantly more products associated with the brands than those in the non-HFSS group (3·76; 95 % CI, 1·90, 5·61; *p* < 0·01). See Figure 5. Across the 24 brands, McDonald’s (HFSS) produced the highest number of product associations (*n* 186, followed by KFC (HFSS, *n* 162), Adidas (non-food, *n* 157) and Cadbury (HFSS, *n* 155)). Almost all HFSS brands produced higher numbers of product associations compared to non-HFSS brands. See Figure 6.

#### Brand recall

**Figure 7.**
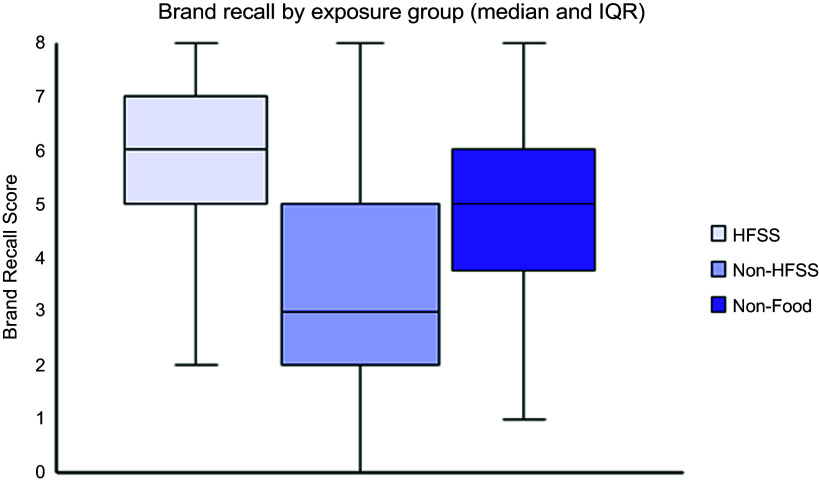
Boxplots showing median brand recall scores for each group.

Brand recall scores significantly differed between advertising exposure groups (H(2), 47·6; *p* < 0·01). Participants in the HFSS group recalled significantly more brands (median = 6, IQR = 5–7) that they were exposed to compared to the non-food (median = 5, IQR = 4–6, Z, 3·74; *p* < 0·01) and non-HFSS groups (median = 3, IQR = 2–5, Z, 6·89; *p* < 0·01). Participants in the non-food group also recalled significantly more brands than the non-HFSS group (Z, 3·26; *p* < 0·01). See Figure 7.

## Discussion

This study provides novel experimental evidence on the impacts of brand advertising using outdoor advertising stimuli on adult food-related outcomes. Findings demonstrated that exposure to HFSS brand advertising had no significant effect on craving or purchase intention for HFSS foods relative to non-HFSS or non-food advertising exposure. However, participants exposed to HFSS brand advertising were significantly more likely to recognise and recall advertised brands compared to participants exposed to non-HFSS and non-food brand advertising. Additionally, participants in the HFSS group were able to associate a larger number of products with the brands they were exposed to, compared to those in the non-HFSS and non-food groups.

The lack of observed effect of HFSS food advertising on affective or behavioural outcomes such as craving or purchase intention among adults is mostly consistent with existing research^(36)^. While robust effects of food advertising on food-related behaviours among children and adolescents have been observed extensively^(3,36,37)^, only a handful of studies have observed effects in adults. Such studies found effects on immediate intake post-advertising exposure in laboratory settings but is predominantly in relation to television advertising^(36)^. Indeed, for children, studies show impacts on recall, recognition, attitudes, preferences, purchase intent and intake for a variety of marketing formats, including via traditional and digital media.

Existing research into HFSS brand advertising *specifically* is limited. A recent systematic review and meta-analysis found that food brand marketing can influence children and adults’ preference, choice and purchase intent, while evidence for an effect on consumption was unclear^(25)^. Notably, the quality of the evidence was mixed, and most synthesised studies examined food packaging, with none testing the impacts of outdoor brand advertising specifically, which was used as stimuli in the current study.

Our findings have important implications for food marketing policy. Firstly, they suggest that brand advertising alone is still memorable and elicits associations with products sold by the brand. This supports the notion that brand advertising should not be exempt from food marketing restrictions, as even without featuring an identifiable HFSS product, it may still enhance recall, recognition and associations with consumable products. This is unsurprising considering that HFSS brands have invested years and funds to establish highly recognisable identities and often encompass more extensive sub-brands and product portfolios than non-HFSS or non-food brands, meaning that a single brand cue can activate a wider network of product associations. For example, McDonald’s, the largest spender on outdoor advertising in 2024 (£86 million)^(15)^, is recognisable simply from its golden arches, having depicted them in isolation for previous advertising campaigns^(38)^. As such, our findings likely reflect the cumulative impact of sustained marketing and brand familiarity, whereby HFSS brands can readily trigger multiple product associations even in the absence of specific products. Consequently, continued promotion of HFSS brands, whether through brand advertising or featuring non-HFSS products in their range, is still likely to elicit associations with HFSS products, underscoring that the influence of HFSS marketing extends beyond individual products and may not be mitigated by product-level restrictions alone.

Importantly, recall and recognition are the first steps in the hierarchy of food marketing effects among children, and it is theorised that repeated exposure over time can impact food attitudes, purchase, consumption and, in the long-term, diet-related disease^(37)^. This hierarchy of effects – from recall to food purchase and consumption via more positive attitudes towards unhealthy foods – has previously been evidenced cross-sectionally among adolescents in response to food marketing in digital media^(39)^. Therefore, it is possible that a single exposure alone was not sufficient to elicit higher-level responses such as craving and purchase intention. Secondly, outdoor brand advertising materials were used as stimuli in this study. Therefore, findings imply that outdoor brand advertising stimuli has the potential to trigger HFSS food recall, recognition and product associations. This supports the need to consider more comprehensive outdoor food advertising restrictions, including for brand advertisements, which are not currently implemented on a national scale in England and are exempt from local restrictions.

The current study has several strengths, including its robust randomised controlled trial design, novel experimental approach to assessing the impacts of brand advertising via outdoor advertising stimuli and examination of effects among adults, who are an under-researched group in the domain of food marketing effects^(36)^. However, there are limitations to be acknowledged. Craving and purchase intention were measured using abbreviated versions of existing tools, which may have been an oversimplification. Relatedly, composite scores were used to measure craving and purchase intention, which may have obscured more specific patterns in the data (e.g. craving/purchase intention may have been significant for specific foods, such as chocolate). Moreover, the time each participant spent looking at the brand advertisements was not controlled, and therefore, it is possible that there was (i) inconsistency between participant exposure duration and (ii) insufficient exposure duration to impact behavioural outcomes, such as purchase intention (the average exposure time among studies in a meta-analysis showing an effect was approximately 5 min^(40)^). While we tried to ensure high levels of familiarity across all brands by selecting only those listed in the top 100 UK brands or with 90 %+ recognition in a YouGov survey, we were not able to assess familiarity of brands among study participants without alerting them to the nature of the study. Therefore, it is possible that some participants were unfamiliar with brands rather than unable to recall them.

It is recommended that future research examines the impact of HFSS brand advertising on children and adolescents, and relative to advertising which features an identifiable product, so that specific comparisons can be drawn to inform policy. Further research on the impacts of outdoor brand advertising specifically would also be beneficial. Immersive technology (e.g. virtual reality headsets) could be utilised to create a more realistic outdoor advertising exposure scenario and to optimise the real-world generalisability of findings. Longitudinal research would be informative to ascertain effects of repeated exposure to HFSS food advertising, as is typical in the real world.

## Conclusion

This randomised controlled trial provides novel evidence on the impact of HFSS brand advertising using outdoor advertising stimuli on adult food-related outcomes. Exposure to HFSS brand advertising prompted recognition and recall of advertised HFSS brands and triggered associations with products offered by the brand. This suggests that HFSS brand advertising, despite not featuring specific products, may still have notable effects on recall, recognition and HFSS brand and product associations, which has important implications for food marketing policy.

## Supporting information

10.1017/S1368980026103115.sm001 Ferneyhough et al. supplementary material Ferneyhough et al. supplementary material

## Supplementary material

For supplementary material accompanying this paper visit https://doi.org/10.1017/S1368980026103115.

## Table 1. Long description

A table with two rows and two columns detailing the inclusion and exclusion criteria for a study. The inclusion criteria are: UK adults aged 18 or older with internet access and able to provide informed consent. The exclusion criteria are: individuals with medically restrictive diets, including pregnant and breastfeeding women, diabetics, and those with Coeliac disease, and individuals who cannot read written English.

Navigate back to Table 1..

## Table 2. Long description

A table comparing characteristics of participants by group. The table has 18 rows and 7 columns. The columns are labeled Characteristics, High in fats, salt and sugar (n 67), Non-high in fats, salt and sugar (n 67), and Non-food (n 68). The rows are labeled Sex, Age in yrs, mean (sd), Baseline hunger, mean (sd), BMI, Ethnicity, and Education. Each row contains data for the categories Male, Female, Age in yrs, mean (sd), Baseline hunger, mean (sd), BMI in kg/m^2, mean (sd), Underweight (<18.5 kg/m^2), Healthy weight (≥18.5 & <25 kg/m^2), Overweight (≥25 & <30 kg/m^2), Obesity (≥30 kg/m^2), BMI missing values, BMI anomalies removed, White, Asian or Asian British, Black, Black British, Caribbean, or African, Mixed or multiple ethnic, Other, Ethnicity missing values, Primary education, Secondary education, Undergraduate degree, Masters degree, Doctorate, Education missing values, None, Vegetarian, Pescetarian, Vegan, Religious dietary practices, Other, Dietary preferences missing values. The table provides detailed data for each category and group.

Navigate back to Table 2..

## Table 3. Long description

A table with 10 rows and 5 columns. The columns are labeled Variable, Level, Coefficient, SE, 95% CI, and P-value. The table presents data on the associations of various factors with purchase intention for high in fats, salt and sugar foods. The factors include advertising exposure, age, baseline hunger, sex, BMI, education, ethnicity, and dietary preference. Each factor has different levels with corresponding coefficients, standard errors, confidence intervals, and p-values. Notable trends include significant associations with age and ethnicity, while other variables show non-significant associations.

Navigate back to Table 3..

## Table 4. Long description

A table with 10 rows and 5 columns showing the associations of various factors with craving for high in fats, salt, and sugar foods. The columns are labeled Variable, Level, Coefficient, SE, 95% CI, and P-value. The table includes data on advertising exposure, age, baseline hunger, sex, BMI, education, ethnicity, and dietary preference. Each row provides specific levels within these variables, along with their corresponding coefficients, standard errors, confidence intervals, and p-values. Notable trends include significant associations for sex, baseline hunger, and age with craving for high in fats, salt, and sugar foods.

Navigate back to Table 4..
