# Supplementary material for: Are we Lovin’ It? A single blind randomised controlled trial to investigate the influence of high in fats, salt and sugar food brand advertising on cognitive, affective and behavioural intention outcomes in UK adults
Source: Public Health Nutr. 2026 Jul 20;29(1):e163. doi: 10.1017/S1368980026103115 (PMC13434566; doi:10.1017/S1368980026103115)
Supplement: Supplementary file 1 [file S1368980026103115sup001.docx]

**Supplementary Material**

Table of Contents

[Supplementary Material A: Included brand advertising exposures for each group	1](#_Toc272935869)

[Supplementary Material B. Brand recall scoring system	4](#_Toc277606858)

[Supplementary Material C. OpenEpi output for full power analysis	8](#_Toc701750669)

[Supplementary Material D. Main analysis showing comparison of regression models including variance of predicted means for cravings.	9](#_Toc1615159478)

[Supplementary Material E: Multivariable models.	11](#_Toc1121408904)

# **Supplementary Material A:** Included brand advertising exposures for each group

Note: image size has been reduced significantly to fit in the appendix. Image size was consistent for all advertisements across each group.

HFSS advertisements:

Non-HFSS advertisements:

Non-Food advertisements:

# **Supplementary Material B.** Brand recall, recognition and product association scoring system

The table below shows the complete range of organic answers provided by participants regarding brand recall and recognition. Correct answers were scored 2 points, with partially correct and incorrect answers being scored 1 point and 0 points respectively.

Typos were accepted within correct answers where the brand name was close beyond reasonable doubt to being correct or within one letter away on a QWERTY keyboard. Well-known sub-brands were also recorded as correct answers (e.g., Dairy Milk was deemed a correct answer for Cadburys). Non-English responses were deemed incorrect.

The table below provides transparency on decision making.

|  | **Brand** | **Answers deemed correct** | **Answers deemed partially correct** | **Answers deemed incorrect** |
| --- | --- | --- | --- | --- |
| **HFSS** | **McDonalds** | Mcdonalds, MacDonalds, Macdonald, Macdonalds, McDonald's, Mac Donalds, McDonald, Mac Donald, McDonalds, MacDonald's, Mc Donalds, Mc Donalds, McDonald's |  |  |
|  | **Greggs** | Greggs, Gregs, Greg's, Greggs, Greegs |  | Bank, BT |
|  | **Pepsi** | Pepsi, Pepsie, Diet Pepsi, I think it might be Pepsi Cola, Peosi | Coca cola |  |
|  | **Cadburys** | Cadburys, Cadbury, Cadburys dairy milk, Cadbury's, Cadbury's chocolate  Cadburies, Dairy milk, Dairy milk, Dairy Milk Chocolate |  |  |
|  | **Dominos** | Dominos, dominoes, Dominoes, Dominos, Dominoes Pizzas, Domino's, Dominoes pizza, Dominos Pizza, Domino's Pizzas, Domino Pizza, Doninoes  Domino |  |  |
|  | **KFC** | KFC, Kentucky fried chicken,  K F C, Kentucky Fried Chicken | Kentucky |  |
|  | **Starbucks** | Starbucks, starbucks, Starbuck, Starbuck's, STARBUCKS |  | Costa Coffee, Startbags |
|  | **Walls** | Walls, walls, Walls ice cream,  Walls', Walls ice cream, Walls Icecream |  | Nestle |
| **Non-HFSS** | **Green Giant** | Jolly Green Giant, Green Giant,  Green giant, Sweetcorn green giant,  Green giant, Jolly green giant | Sweetcorn, sweetcorn, sweetcorn guy, Seeetcorn, Little green giant | Nibblets sweet, Heinz,  Geant Vert (don't know the name in English sorry) |
|  | **Innocent** | Innocent, Innocent, Innocent smoothie, Inoccent, Innocent drinks, Inocent, Innocent smoothies, Innocence | Honest, Smoothies |  |
|  | **Arla** | Arla, Arla (Dairy) | Arlo | Flora, Clover, Buttercup, Margarine, |
|  | **Princes** | Princes, Princess, Prince, Princes', Princes  Princes Tuna, Prince's |  | Telegraph news paper, Lurpak |
|  | **Del Monte** | Del monte, Del Monte, Delmonte |  | Dolmio, curry, Dolce something |
|  | **Quaker** | Quaker Oats, Quakers, Quaker  Quaker oats, Quaker  Quacker, Porridge oats,  Quakers, Quake oats, Quacker oats |  | Scotts, Jacob Oats |
|  | **Old El Paso** | old el paso, El Paso, El Paso, Olde el paso, El passo, Old El Paso, El.paso  Old el passo, Old Ellpasso, El del paso, Old El Pasco, El do paso, El Passo | Mexican Food, El paco | Can’t remember |
|  | **Evian** | Evian, Evian Water |  | Alpen, Volvic, Everest |
| **Non-Food** | **Adidas** | Adidas, Adidas, ?Adidas, Addidas, Addidas, Sdidas |  | Reebok, Nike |
|  | **Google** | Google, ?Google |  |  |
|  | **Tui** | Tui, Thomas Cook (tui), TUI  Thomas cook tui, TU holidays, Rui |  | Thompson |
|  | **Mastercard** | Mastercard, Marstercard, Master card, Mastacard, Master Card | Credit card | Barclay card, Visa, NATWEST |
|  | **Premier Inn** | Premier Inn, Premier inn, Premier inns | Holiday Inn Something Inn | Travellodge, Travel lodge, Travelodge, Silent night, Travelodge |
|  | **Gucci** | Gucci |  | Chanel, Card phone, Dior, Chanel? |
|  | **Andrex** | Andrex, Andrex?, Andrex toilet roll | Toilet rolls, Andrew | Dulux |
|  | **Fairy** | Fairy, fairy, Faiy liquid, Fairly Liquid |  | Dettol, Granier, Castrol, Vosene, Aerial |

For product associations, general categorical descriptions (e.g. ‘burger’, ‘shoes’, ‘vegetables’) and specific product names (e.g. ‘Big Mac’, ‘Air Max’, ‘Sweetcorn’) were accepted as correct associations. If a participant provided overlapping general/specific answers (e.g. ‘burger’, ‘Big Mac’), both associations were considered correct. Non-product associations (e.g. ‘cheap’) were considered incorrect associations. Products from sub-brands were also categorised as correct product associations.

**Supplementary Material C.** OpenEpi output for full power analysis

# **Supplementary Material D.** Main analysis showing comparison of regression models including variance of predicted means for cravings.

Table 1 shows the consistency in regression models regarding statistical significance. The following page contains screenshots showing the similarity of fitted means between each model.

**Table 1**: Comparison of regression models for advertising exposure group and cravings for HFSS foods

| **Regression Model** | **Coefficient (SE)** | | | | **95% CI** | **P-value** |
| --- | --- | --- | --- | --- | --- | --- |
| **GLM regression** |  | | | | |  |
| **HFSS (intercept)** |  |  |  | 233.65 (25.14) | 184.38, 282.91 | <0.01 |
| **Non-HFSS** |  |  |  | -11.49 (36.7) | -82.57, 59.60 | 0.752 |
| **Non-Food** |  |  |  | -12.73 (35.55 | -82.40, 56.94 | 0.721 |
| **GLM regression following sqrt transformation** | | | | | | |
| **HFSS (intercept)** | 13.59 (0.91) | | | | 11.80, 15.39 | <0.01 |
| **Non-HFSS** | -0.42 (1.32) | | | | -3.00, 2.16 | 0.751 |
| **Non-Food** | -1.08 (1.29) | | | | -3.61, 1.45 | 0.405 |
| **Zero inflated Beta regression: Cravings (bounded between 0 and 1) Mu parameter** | | | | | | |
| **HFSS (intercept)** | -1.14 (0.13) | | | | -1.40, -0.89 | <0.01 |
| **Non-HFSS** | 0.03 (0.18) | | | | -0.32, 0.38 | 0.877 |
| **Non-Food** | -0.05 (0.18) | | | | -0.40, 0.30 | 0.772 |

Screenshots below of fitted means for each model for cravings. Fitted means between Original GLM regression (top), square root transformed regression (middle) and beta regression (bottom) for cravings:

Similarly to the regression analysis for cravings, Table 2 shows the consistency in regression models regarding statistical significance for purchase intention. The following page contains screenshots showing the similarity of fitted means between each model for purchase intention.

**Table 2:** Comparison of regression models for advertising exposure group and purchase intention for HFSS foods

| **Regression Model** | **Coefficient (SE)** | | | | **95% CI** | **P-value** |
| --- | --- | --- | --- | --- | --- | --- |
| **GLM regression** |  | | | | |  |
| **HFSS (intercept)** |  |  |  | 234.89 (23.24) | 189.34, 280.44 | <0.01 |
| **Non-HFSS** |  |  |  | -51.91 (33.53) | -117.63, 13.81 | 0.123 |
| **Non-Food** |  |  |  | 31.21 (32.87 | -33.20. 95.63 | 0.343 |
| **GLM regression following sqrt transformation** | | | | | | |
| **HFSS (intercept)** | 13.59 (0.83) | | | | 11.97, 15.21 | <0.01 |
| **Non-HFSS** | -1.35 (1.19) | | | | -3.68, 0.99 | 0.261 |
| **Non-Food** | 1.141 (1.17) | | | | -1.15, 3.43 | 0.330 |
| **0 Inflated Beta regression (bounded between 0 and 1) Mu parameter** | | | | | | |
| **HFSS (intercept)** | -1.37 (0.13) | | | | -1.63, -1.11 | <0.01 |
| **Non-HFSS** | 0.10 (0.18) | | | | -0.25, 0,45 | 0.573 |
| **Non-Food** | 0.16 (0.18) | | | | -0.19, 0.50 | 0.373 |

Screenshots below of fitted means for each model for purchase intention. Fitted means between Original GLM regression (top), square root transformed regression (middle) and beta regression (bottom).

# **Supplementary Material E:** Multivariable models.

**Table 3**: Associations between variables and purchase intention for HFSS foods, including covariates

| **Variable** | **Level** | **Coefficient (SE)** | **95% CI** | **P-value** |
| --- | --- | --- | --- | --- |
|  |  |  |  |  |
| **Advertising exposure** | HFSS (Intercept) | 472.46 (51.35) | 371.82, 573.10 |  |
|  | Non-HFSS | -45.83 (30.42) | -105.45, 13.78 | 0.134 |
|  | Non-Food | 15.45 (29.85) | -43.05, 73.96 | 0.605 |
| **Age** |  | -4.24 (0.79) | -5.78, -2.69 | <0.01* |
| **Ethnicity** | White | -39.78 (42.30) | -122.70, 43.13 | 0.348 |
| *Statistically significant (*p*<0.05)  Ethnicity group condensed as original category had < 5 data points | | | | |

**Table 4**: Associations between variables and craving for HFSS foods, including covariates

| **Variable** | **Level** | **Coefficient (SE)** | **95% CI** | **P-value** |
| --- | --- | --- | --- | --- |
|  |  |  |  |  |
| **Advertising exposure** | HFSS (Intercept) | 360.16 (49.36) | 263.63, 456.70 |  |
|  | Non-HFSS | -8.56 (33.03) | -73.30, 56.18 | 0.796 |
|  | Non-Food | -15.39 (32.33) | -78.75, 47.97 | 0.635 |
| **Age** |  | -3.97 (0.84) | -5.63, -2.32 | <0.01* |
| **Baseline hunger** |  | 1.46 (0.53) | 0.43, 2.49 | <0.01* |
| **Sex (Male)** | Male | 85.12 (30.86) | 24.64, 145.60 | <0.01* |
| *Statistically significant (*p*<0.05) | | | | |
